# Celastrol induces apoptosis in hepatocellular carcinoma cells via targeting ER-stress/UPR

**DOI:** 10.18632/oncotarget.21750

**Published:** 2017-10-10

**Authors:** Bo Ren, Hui Liu, Hang Gao, Shutong Liu, Zehui Zhang, Andrew M. Fribley, Michael U. Callaghan, Zhixiang Xu, Qinghua Zeng, Yulin Li

**Affiliations:** ^1^ The Key Laboratory of Pathobiology, Ministry of Education, Jilin University, Changchun 130021, China; ^2^ Pathology, The Second Affiliated Hospital of Zhejiang University School of Medicine, Hangzhou 310009, China; ^3^ Carman and Ann Adams Department of Pediatrics, Division of Hematology/Oncology, Wayne State University, Detroit, MI 48201, USA; ^4^ Molecular Therapeutics Program, Karmanos Cancer Institute, Detroit, MI 48201, USA; ^5^ Division of Hematology/Oncology, Department of Medicine, University of Alabama at Birmingham, Birmingham, AL 35294, USA

**Keywords:** hepatocellular carcinoma, ER stress, autophagy, celastrol, apoptosis

## Abstract

Hepatocellular carcinoma (HCC) is one of the most serious and deadly diseases worldwide with limited options for effective treatment. Biomarker-based active compound targeting therapy may shed some light on novel drugs for HCC. The endoplasmic reticulum (ER) stress and unfolded protein response (UPR) play important roles in the regulation of cell fate and have become novel signaling targets for the development of anticancer drugs. Celastrol, a triterpene from traditional Chinese medicine, has been reported to possess anti-tumor effects on various cancers. We, along with several other research groups, have recently reported that UPR was induced by celastrol in several different cancers, including hepatocellular carcinoma. However, UPR status in HCC still remains unclear. The role of ER stress and autophagy in response to celastrol also has yet to be elucidated. Our results demonstrated that celastrol could cause G2/M phase rest and inhibit proliferation in HepG2 and Bel7402. Exposure to celastrol resulted in the activation of the intrinsic apoptotic pathway, via ER stress and the UPR. In murine syngeneic model studies celastrol inhibited H22 tumor growth via the induction of ER stress and apoptosis. Our study suggests that celastrol is a potential drug for HCC therapy via targeting ER-stress/UPR.

## INTRODUCTION

Hepatocellular carcinoma (HCC) is a very common form of cancer and is associated with an extremely poor prognosis; HCC is the third most common cause of cancer-related deaths worldwide [1, 2]. HCC is particularly prevalent in Asia and sub-Saharan Africa where 80% of worldwide cases occur [2, 3]. However, new evidence suggests that the incidence of HCC is increasing in some Western countries [2, 4]. Unfortunately, more than 80% of HCC patients present with advanced disease, and the treatment options available to these patients are very limited and the prognosis is dire [5]. Although there are a variety of drugs (e.g., sorafenib) used in the treatment of the disease, many of them are quickly rendered ineffective due to resistance [6]. Developing anticancer drugs from bio-renewable traditional Chinese medicine is an alternative approach to identify novel potential therapeutics for HCC.

Celastrol is a pharmacologically active pentacylclic triterpenoid compound originally identified from the Thunder of God Vine root (*Tripterygium wilfordii* and *Celastrus regelii*), and has been used as a natural remedy for inflammatory conditions and autoimmune diseases for years [7]. Recently, it was regarded as one of the most promising medicinal molecules from plant extracts of traditional medicines [8]. Many studies have indicated that celastrol, as a cytotoxic agent, induces apoptosis in various kinds of cancer cells including HCC cells [8–15]. Studies have also shown that celastrol inhibited TNF-mediated NF-κB signaling pathway to promote TRAF2 associated apoptosis in leukemia cells [16]. A recent study showed celastrol could induce apoptosis in HCC cells by activating Noxa and modulating Mcl-1 [17]. Celastrol was also regarded as a potential proteasome inhibitor in glioblastoma and prostate cancer cells [18]. We have recently reported that celastrol induced endoplasmic reticulum (ER) stress and apoptosis in head and neck squamous cell carcinoma and could not inhibit the 26S proteasomal degradation pathway in biochemical assays [19].

The ER functions to regulate multiple cellular processes such as protein folding, post-translational modification and trafficking, and Ca^2+^ signaling in addition to maintaining the cellular homeostasis of cells and the quality control of folded proteins [20]. To restore homeostasis in the face of extracellular stress, cells have adapted a signaling mechanism known as the unfolded protein response (UPR) [21]. Several pathophysiological conditions such as abnormal protein synthesis, nutrient deprivation, virus infection, Ca^2+^ flux imbalance, and redox imbalance have been shown to promote activation of the UPR [22]. This adaptive UPR is initiated by the activation of IRE1α and the subsequent splicing of *XBP1 (XBP1s)*, which leads to the transcription of a host of chaperones and other enzymes that return to the ER lumen in an attempt to restore homeostatic peptide processing. However, under conditions where the UPR is not able to restore homeostatic protein folding, or where the stress is robust or persistent, the UPR culminates in a cascade of events that ultimately leads to cell death [23]. In mammalian cells UPR signaling is initiated by three endoplasmic reticulum transmembrane protein sensors: inositol-requiring enzyme 1α (IRE1α), double-stranded RNA dependent protein kinase-like ER kinase (PERK) and activating transcription factor 6 (ATF6) [24]. IRE1α is a Ser/Thr protein kinase and endoribonuclease that catalyzes the unconventional processing of the mRNA encoding the transcriptional factor X-Box binding protein 1 (XBP1) [25].

Conditions of prolonged ER-stress lead to the transcription and post translational activation of BH3-only proteins (e.g., NOXA and PUMA), trigger the initiation of ER stress-mediated apoptosis [26, 27]. The exact mechanisms by which UPR signaling components initiate apoptosis have not been completely elucidated. ATF4, p53 and possibly CHOP regulate PUMA and NOXA expression under chronic ER stress [28, 29]. In human melanoma cells, activation of the ATF6 and IRE1α signaling pathways are all involved in the transcriptional upregulation of Bcl-2 and Mcl-1 expression in tunicamycin (interferes with n-linked glycosylation) and thapsigargin (leads to rapid irreversible Ca^2+^ flux from the ER lumen to cytosol) induced UPR [30, 31].

When misfolded proteins cannot be degraded by the proteasome, the UPR may also upregulate autophagy machinery [32]. Autophagy is a catabolic process involving lysosomal turnover of proteins and organelles for maintenance of cellular homeostasis and mitigation of metabolic stress. Autophagy defects promote cancer progression in association with oxidative and ER stress, DNA damage accumulation, genomic instability and persistence of inflammation, while functional autophagy enables cancer cell survival under stress and likely contributes to treatment resistance [33]. The subsequent activation of autophagy recycles and degrades proteins to limit ER stress and provide nutrients and metabolites to the cell. When cellular stress conditions are prolonged or excessive, autophagy can promote caspase-independent cell death [34]. Low levels of autophagy promote survival, but if unbalanced tips the scale towards cell death. This duality complicates its role in the treatment of cancer, and more knowledge about activation, function, and effects of autophagy is crucial in order to utilize this pathway to improve cancer therapy [35].

In this study, we investigated the potential anticancer abilities of celastrol in a pair of human HCC cells. Our findings demonstrate that celastrol induced apoptotic ER stress signaling and autophagy and suppressed HCC cell proliferation. Ourstudy additionally revealed that celastrol induced apoptosis in cultured HCC cells was significantly diminished by the addition of the chemical chaperone tauroursodeoxycholate (TUDCA). Our findings highlight that celastrol increased ER-tress in HCC, and underscore a growing body of literature describing the potential of its use therapeutically for HCC and other cancers.

## RESULTS

### Celastrol alters cell viability, and growth kinetics of HCC cells

**Figure 1:**
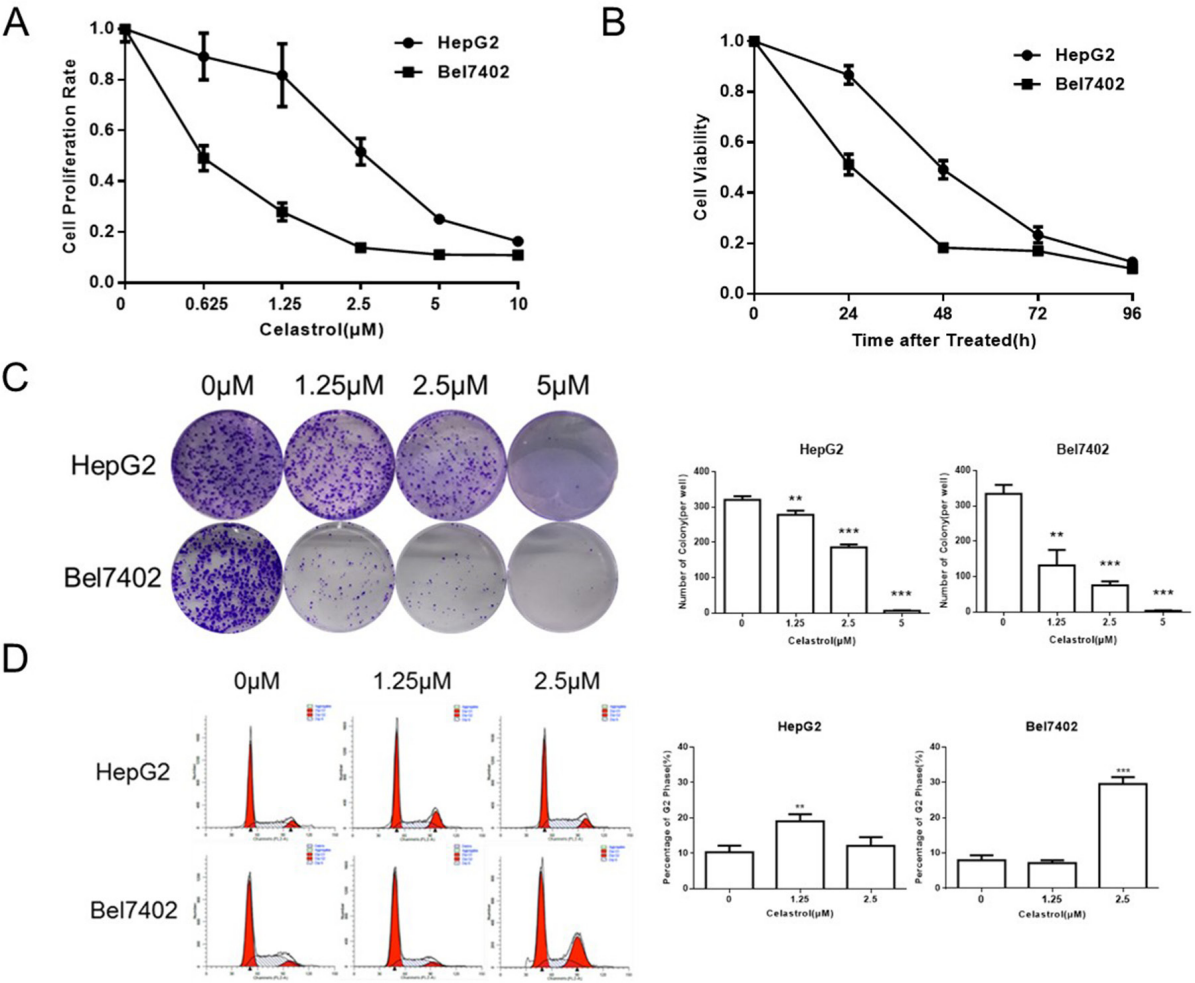
Celastrol inhibited proliferation in HCC cell lines **(A, B)** CCK-8 assays were performed in HepG2 and Bel7402 cells after exposure to increasing doses of celastrol for 24 hours, and after exposure to 1.25 μM celastrol for 24-96 hours. **(C)** Clonogenic cell survival assays in HepG2 and Bel7402 treated for 1 hour with 1.25, 2.5 and 5 μM of celastrol prior to washout, ^*^P < 0.05, student's t-test. **(D)** HepG2 and Bel7402 cells were cultured with 1.25 and 2.5 μM celastrol for 24 hours. The cell cycle was determined by flow cytometry after fixation and propidium iodide staining, ^*^P < 0.05, student's t-test.

Proliferation assays performed with HepG2 and Bel7402 cells exposed to celastrol (0.625 - 10μM) for 24h revealed dose-dependent reductions in cell proliferation (Figure 1A). The rate of proliferation (inhibition) was also determined over a 96h period with 1.25μM (Figure 1B). Celastrol had both dose- and time-dependent antiproliferative effects in Hep G2 and Bel7402 cells. Colony-forming assays were performed to test the effect of celastrol on growth kinetics; HepG2 and Bel7402 cells were seeded at a low density and treated with increasing concentrations of celastrol. Cells were exposed to celastrol for two hours, washed and replenished with drug-free medium, and incubated for 10 days. Celastrol significantly decreased (P<0.01) the number of colonies formed in both HepG2 and Bel7402 cells (Figure 1C). The effect of celastrol on cell cycle distribution of HepG2 and Bel7402 cells was studied to elucidate the mechanism of the anticancer effect. Celastrol led to G2/M phase arrested, in a dose dependent manner, in both HepG2 and Bel7402 cells (Figure 1D). These results were consistent with observed reductions in cell viability. This accumulation of cells in G2/M phase also coincided with the occurrence of a sub-G1 population indicative of an increasing population of apoptotic cells following exposure to celastrol.

### Celastrol induced apoptosis in HCC cells

**Figure 2:**
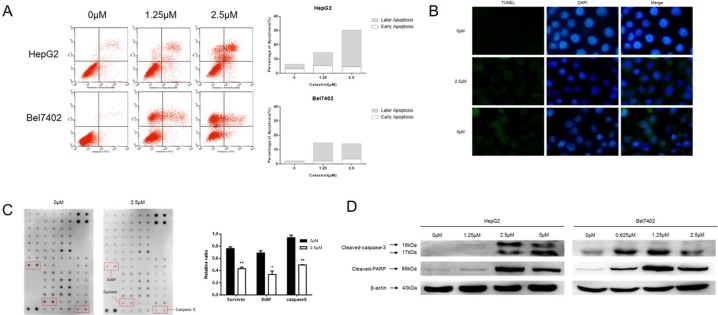
Celastrol induced apoptosis in HCC cells **(A)** HepG2 and Bel7402 cells were treated with 1.25 and 2.5 μM celastrol for 24 hours. Apoptosis was measured via PI/Annexin V-FITC staining and quantified by flow cytometry. **(B)** Apoptotic cell death in HepG2 by celastrol treatment was demonstrated by TUNEL assay, and representative micrographs are shown (magnification, ×400). **(C)** Original figure of Human Apoptosis Antibody array (abcam 134001) and graph of protein expression detected by array. ^*^P < 0.05 compared with the equimolar DMSO, student's t-test. **(D)** Immunoblot analysis of whole-cell lysates from HepG2 and Bel7402 cells treated with celastrol and probed with monoclonal antibodies for cleaved caspase 3 and cleaved PARP. For immunoblot analysis, each membrane was stripped and re-probed with monoclonal β-actin. “0 μM” indicates equimolar DMSO control.

In order to test whether the inhibition effect of celastrol on cell proliferation and cell cycle arrest was due to the activation of apoptosis HepG2 and Bel7402 cells, Annexin-V staining was performed (Figure 2A). As the concentration of celastrol increased, so did the population of apoptotic cells; both early and late apoptotic populations were observed. Challenging the cells with celastrol also led to a marked increase in exposed 3′ OH groups as revealed by TUNEL and DAPI staining which is a typical morphological feature of apoptotic cells (Figure 2B). Human apoptosis antibody array of HepG2 was performed with cells treated with 2.5μM celastrol; the procaspase8 and anti-apoptotic proteins survivin and XIAP were decreased significantly (Figure 2C). We have got similar results in apoptosis antibody array of Bel7402 (Supplementary Figure 1). And the apoptosis associated proteins, such as active caspase-3 and PARP were tested by immunoblot analysis in HepG2 and Bel7402 cells (Figure 2D). There was a robust increase in the activated forms of both of these transcripts, in a dose-dependent manner. These results indicate that celastrol induced cell death through activating the intrinsic (mitochondrial-mediated) apoptotic pathway.

### Celastrol induced autophagy in HCC cells

**Figure 3:**
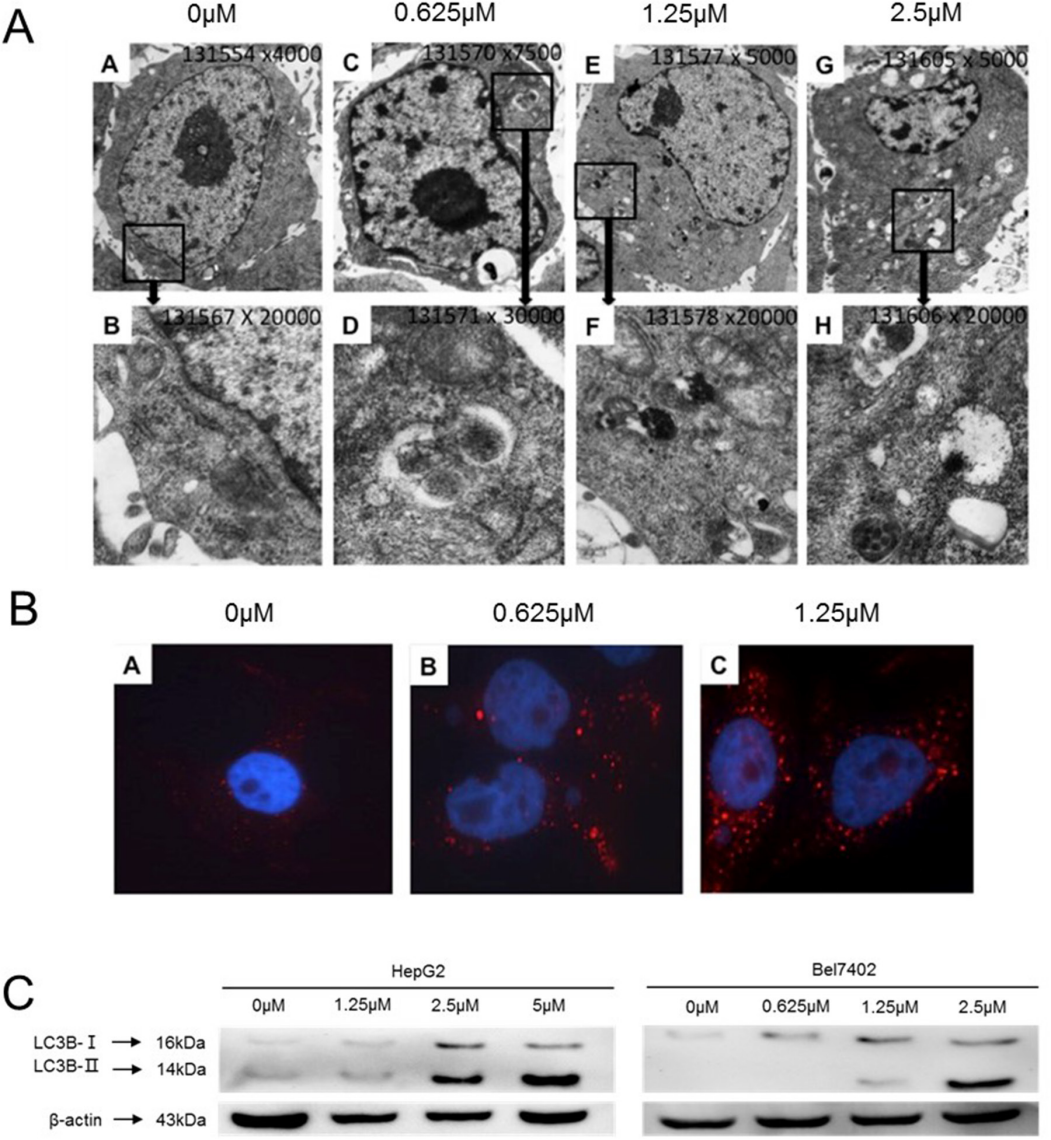
Celastrol induced autophagy in HCC cells **(A)** Electron microscopy images showing ultrastructural features of a representative control cell and the morphological features of autophagy in Bel7402 cells treated with 0.625 μM, 1.25μM and 2.5μM celastrol for 24 h. Cells were observed at different magnifications (x 5 000 and x 20 000). **(B)** Bel7402 cells were fixed, permeabilized, and successively incubated with anti-LC3B antibodies and a secondary antibody conjugated Alexa Fluor^®^ 647. The nuclei were stained with DAPI. **(C)** LC3B expression in HepG2 and Bel7402 cells after treatment with 1.25 and 2.5 μM celastrol for 24 hours.

There have been credible reports in the literature demonstrating the ability of celastrol to induce autophagy in a wide-variety of cancer cells [35, 36]. In the current study, electron microscopy was employed to observe the subcellular structure of Bel7402 cells treated with increasing concentrations of celastrol for 24h (Figure 3A). The number of observed autophagosomes increased with celastrol concentration. Immunofluorescent observation using LC3B labeled with Alexa Fluor 647 similarly demonstrated that red fluorescent particles in the cytoplasm of Bel7402 cells increased with celastrol dose (Figure 3B). Immunoblot analysis revealed that LC3B-II (cleaved) expression also increased with 2.5μM celastrol treatment in HCC (Figure 3C). The above results suggest that celastrol induced autophagy in HCC cells, which is consistent with similar studies in other pre-clinical cancer models [36].

### Celastrol induced ER stress in HCC cells

**Figure 4:**
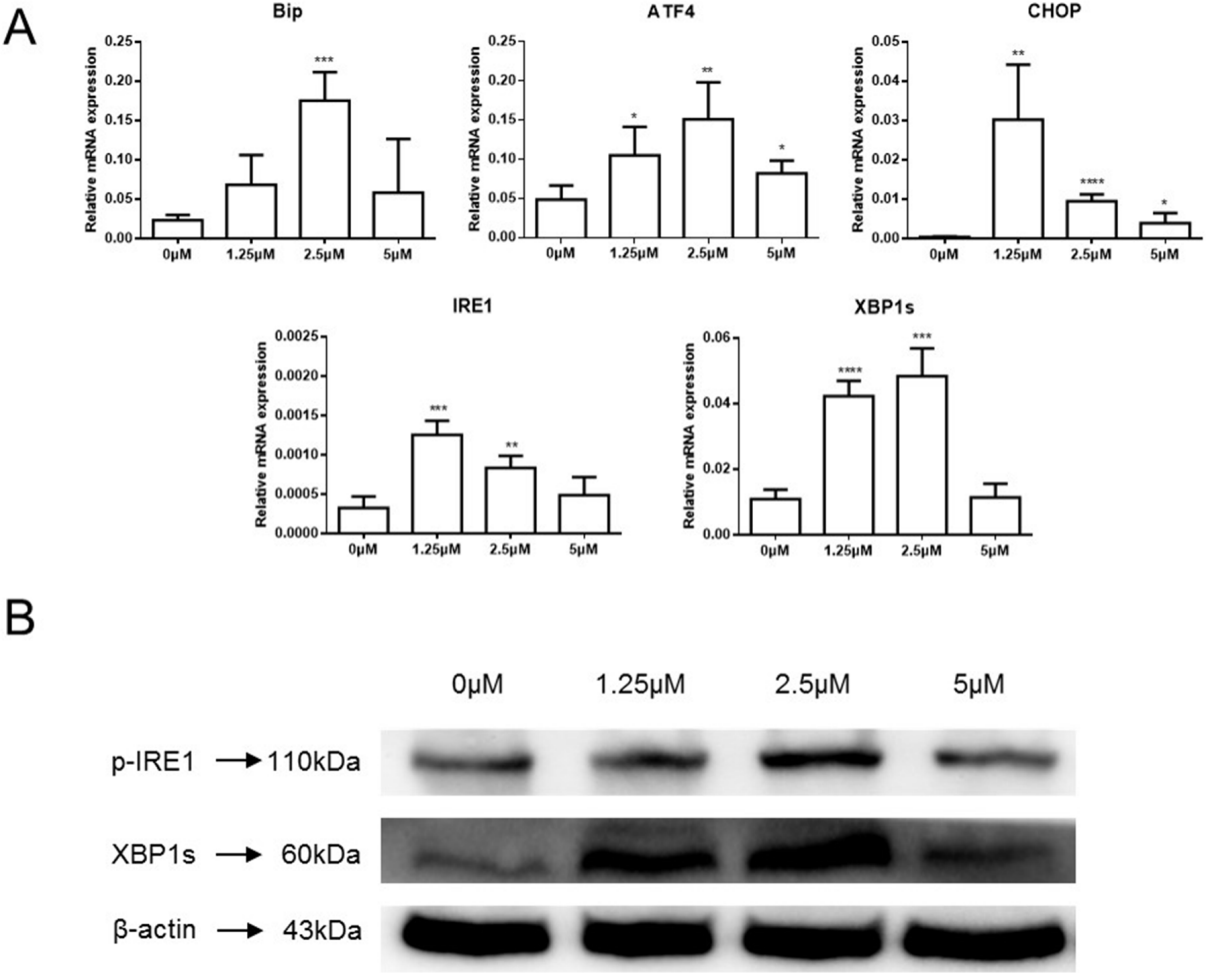
Celastrol induced ER stress in HCC cells **(A)** ER stress related gene expression in HepG2 cells treated with celastrol were analyzed by real-time qPCR, ^*^P < 0.05, student's t-test. **(B)** Immunoblot analysis of whole-cell lysates from HepG2 cells treated with celastrol and probed with monoclonal antibodies for p-IRE1 and spliced XBP1. For immunoblot analysis, each membrane was stripped and re-probed with monoclonal β-actin. “0 μM” indicates equal molar DMSO control.

Members of our group have reported that celastrol could induce ER stress, the UPR and apoptosis in head and neck squamous cell carcinoma [18]. The mRNA level of ER stress- and UPR-related proteins was examined in the current study to determine if celastrol treatment could challenge the secretory system. *GRP78/BiP*, *ATF4*, *CHOP*, *IRE1α* and the spliced form of *XBP1* (*XBP1s*) significantly increased in HepG2 cells exposed to celastrol (Figure 4A). Immunoblot results also showed increased phosphorylation of IRE1α and XBP1s, especially at 2.5μM (Figure 4B). These results indicate that celastrol caused ER stress in HCC cells, furthermore, the induction of CHOP mRNA and protein transcripts suggests that celastrol induced ER stress might be an important feature of celastrol-induced apoptosis in HCC cells.

### TUDCA attenuated cell apoptosis in HCC cells induced by celastrol

**Figure 5:**
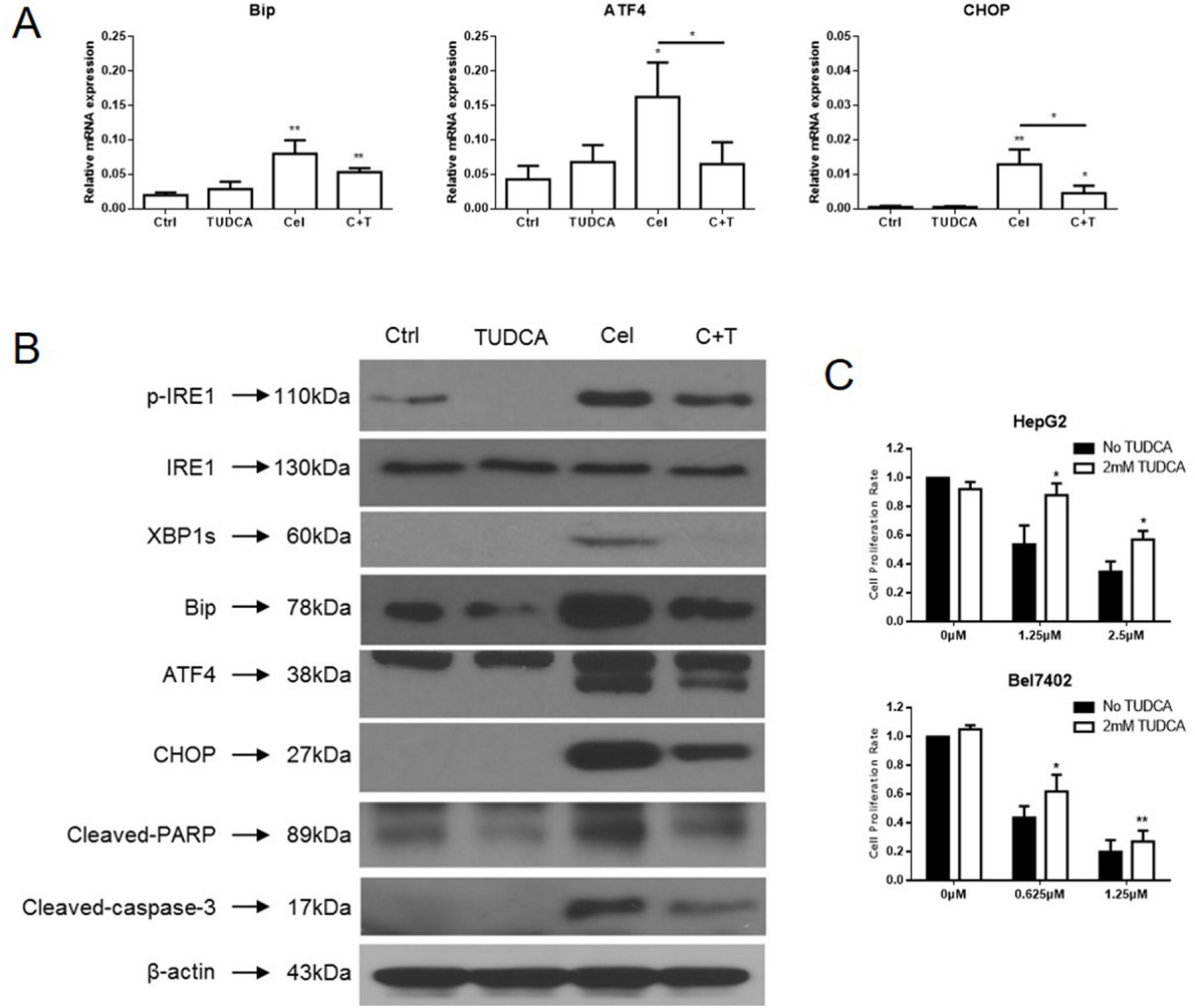
TUDCA relieves celastrol induced ER stress in HCC cells **(A)** ER stress related genes expression in HepG2 cells treated with celastrol and TUDCA were analyzed by real-time qPCR, ^*^P < 0.05, student's t-test. **(B)** Immunoblot analysis of whole-cell lysates from HepG2 cells treated with celastrol and TUDCA probed with antibodies of ER stress and apoptosis. For immunoblot analysis, each membrane was stripped and re-probed with monoclonal β-actin. “0 μM” indicates equal molar DMSO control. **(C)** CCK-8 assays were performed in HepG2 and Bel7402 cells after exposure to a serial dose-response of celastrol with or without 2mM TUDCA for 24 hours, ^*^P < 0.05, student's t-test.

GRP78/BiP, ATF4, and CHOP expression were increased in HCC cells treated with celastrol. TUDCA, a chemical chaperone and ER stress inhibitor, was added into the HepG2 culture medium 45 minutes before the cells were challenged with celastrol. The mRNA levels of ATF4 and CHOP, which belong to the PERK-elF2α-ATF4-CHOP axis of ER stress response, were decreased significantly in HepG2 cells exposed to celastrol in combination with 2mM TUDCA compared to those treated with celastrol alone (Figure 5A). Although the mRNA level of *GRP78/BiP* decreased slightly it was still significantly higher than the untreated group. Similar results were observed with immunoblot analysis of whole cell lysates harvested from celastrol-TUDCA treated cultures. The expression of GRP78/BiP, ATF4, and CHOP were reduced by TUDCA in HepG2 cells exposed to celastrol (Figure 5B). The reduced expression of GRP78/BiP and CHOP suggested that the addition of chemical chaperone might effectively restore global translation. Furthermore, TUDCA reduced celastrol-induced cleavage of procaspase-3 and PARP and attenuated the antiproliferative effect of celastrol on HCC cells in culture (Figure 5C). Overall, these data indicate that TUDCA reduced the celastrol induced ER stress and thereby attenuated apoptotic cell death. TUDCA did not significantly effect the ability of celastrol to induce autophagy (data did not show). These data suggest that the primary anti-tumor effect of celastrol is likely mediated by induction of cellular apoptosis in HCC cells via unregulated ER-stress.

### Celastrol inhibited tumor growth and induced cell apoptosis *in vivo*

**Figure 6:**
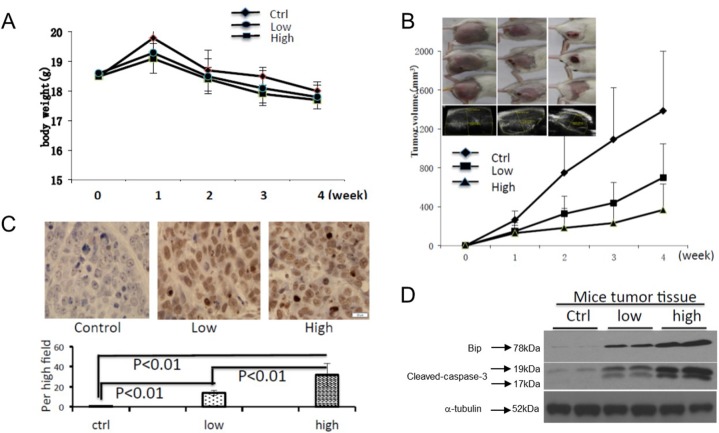
Celastrol inhibited tumor growth and induced cell apoptosis *in vivo* To generate mouse tumor model, 2.0×10^6^ H22 cells were injected subcutaneously in the right flank of BALB/c mice, and then intraperitoneal administration of vehicle or celastrol (1 or 2 mg/kg) daily was started one day after injection. **(A)** The body weight changes; **(B)** representative pictures of tumors in each group, and the tumor volume curve of each group; data are shown as mean ± standard error of the mean; n = 5 for each group, ^*^P < 0.05, student's t-test. **(C)** Mouse tumor sample TUNEL was analyzed using IHC. **(D)** The expression of GRP78/BiP, and activated caspase-3 in mouse tumor samples were analyzed by immunoblot.

Hepatoma H22-bearing mice were used to evaluate the anti-tumor activities of celastrol *in vivo*. Two groups of mice were treated with low or high dosage of celastrol and compared to a control group that received only corn oil vehicle control. Over the course of the experiment the body weights of the animals remained similar (Figure 6A). The tumor volumes were dramatically reduced in mice of treated with either dose of celastrol (Figure 6B, p<0.01). TUNEL immunohistochemical analysis revealed the presence of more apoptotic cells in the tumor tissues of celastrol-treated mice compared to those of the control (Figure 6C). Western immunoblot analysis performed with fresh whole tumor lysates demonstrated increased expression of GRP78/BiP and activated caspase-3 from celastrol treated mice (Figure 6D, p = <0.01), a trend that was even more robust in mice treated with the higher dose of celastrol (p<0.01). These data indicate that celastrol increases ER stress and suggests that xenograft growth is inhibited by the induction of caspase activities.

## DISCUSSION

Accumulating evidence suggests that celastrol has anticancer effects in a wide variety of preclinical cancer models. Celastrol inhibits various cancer cells proliferation and promotes apoptosis in prostate cancer, breast cancer, colon cancer, osteosarcoma and oral squamous cell carcinoma [19, 37–41, 45]. Celastrol can also be used as a sensitizer to enhance the effect of radiotherapy for prostate cancer [42]. In lung cancer cells, celastrol enhanced the effect of chemotherapy with cisplatin [43, 44]. In leukemia cells, celastrol inhibited pro-survival NF-κB signaling and enhanced TNFα-mediated apoptosis [16]. While in osteosarcoma cells, the mitochondrial apoptosis pathway can be activated by celastrol [45]. Furthering the understanding of the molecular mechanism of celastrol will allow researchers and potentially clinicians to better utilize this drug for cancer treatment. In HCC cells, celastrol was reported to inhibit PI3K/Akt signaling pathway through the activation of JNK and ultimately to inhibit cell proliferation by apoptosis [41]. ROS accumulation has also been implicated in celastrol induced JNK activation [37]. Our study suggests that celastrol induces apoptosis via decreased expression of anti-apoptotic protein XIAP and survivin in concert with the activation of caspases in HCC. In the current study celastrol could induce apoptosis in both HCC cell lines examined. Although HepG2 is not generally considered to be a chemoresistant cell line, it should be noted that the IC50 for Bel7402 (∼0.625μM) and HepG2 (∼2.5μM) were significantly different. The range of observed IC50 concentrations for these HCC cell lines is consistent with our previous studies using oral squamous cell carcinoma (OSSC) cell lines [19]. Although the reason HepG2 cells were able to tolerate significantly higher doses of celastrol is not completely clear, it could be due to the slightly increased base line autophagy we observed or differences in, basal levels of ER stress. Also, the three OSCC cell lines with IC50 > 2.5μM (from our previous study) harbored known mutations in TGFβ/SMAD signaling that are known clinically to provide resistance to chemotherapy. Considered together these studies underscore the possibility that unknown genetic variations might contribute to the discrepancy in IC50 values for HCC. Importantly, these differences provide a platform to identify genetic factors that might be used to identify which patients are more likely (or not) to responds to celastrol therapy.

The highly secretory nature of many human cancers and the constant demand for new cell surface and lysosomal proteins leads to chronic ER stress and UPR signaling. The “UPR hypothesis” is predicated on the notion that an ER stressor (i.e., small molecules or natural products) could be delivered systemically to direct cancer cells towards an apoptotic fate, whereas adjacent non-malignant cells (with low or no basal UPR activation) might see the challenge, mount an effective adaptive UPR, and return to homeostasis [46, 47]. Celastrol has been widely reported to inhibit the function of the 26S proteasome, a mechanism known to induce ER stress and activate apoptotic UPR signaling [47–49]. We found that expression of ubiquitinated proteins increased rapidly and dramatically in HCC cells exposed to celastrol (Supplementary Figure 2), similar to previous observations in head and neck squamous cell carcinoma cells [18]. The current study also found that celastrol induced intracellular calcium flux (Supplementary Figure 2), one of the best characterized mechanisms of ER stress. Therefore, it is likely that the ability of celastrol to modulate Ca^2+^ stores plays an important role in the induction of ER stress and the UPR in HCC. The addition of the chemical chaperone TUDCA to celastrol treated cultures led to decreased expression of a series of downstream proteins associated with ER stress, including CHOP, which is regarded as a marker of apoptosis induced by ER stress. The expression level of phosphorylation of IRE1α, XBP1s were also decreased in these cultures. IRE1α-XBP1 is considered to promote an adaptive response that facilitates cell survival during and following ER stress. p38 has been demonstrated to be involved in the post-translational modification of XBP1s, which may be related to the expression level of XBP1s. In the current study p38 was not affected by the ER stress reliever TUDCA [48]. We also observed that celastrol enhanced autophagy in HCC cells. The downstream transcription factor(s) of ER stress and unfolded protein response expression induced autophagy related protein expression [49]. However, in the current analysis, there was not a direct correlation between autophagy and ER stress induced cell death caused by celastrol in HCC cells. Investigating the correlation between autophagy and ER stress caused by celastrol could enable us to understand celastrol anticancer effects.

Considered together, our *in vitro* and *in vivo* experiments demonstrate that celastrol induced apoptosis in HCC cells by inducing ER stress and activating the UPR. The fact that celastrol reduced tumor burden *in vivo* in a dose dependent fashion is especially encouraging, especially since no adverse effects were observed over time even in mice treated at the highest dose. The translation of celastrol to the clinic will depend on myriad factors that are beyond the scope of this study however, we have been able to glean important insight into the potential of using a well-known compound from traditional Chinese medicine to induce ER stress and reduce the proliferation of HCC cells.

## MATERIALS AND METHODS

### Cells and reagents

The hepatocellular carcinoma cell lines HepG2 and Bel7402 were purchased from Cell Bank of Type Culture Collection of Chinese Academy of Sciences, Shanghai Institute of Cell Biology. HepG2 and Bel7402 were grown in RPMI-1640 Medium (Gibco) supplemented with 10% fetal bovine serum (Gemini) and 1% penicillin/streptomycin (Hyclone) were maintained at 37°C in a humidified incubator containing 5% CO_2_. The murine ascetic H22 hepatoma cell line was obtained from the China Center for Type Culture Collection (Wuhan, China). After recovery from frozen stocks, H22 cells were suspended in normal saline and cultured in the peritoneal cavity of the mice. Celastrol (Sigma) was dissolved in DMSO (Sigma) and stored as a 10mM stock solution at −20°C. TUDCA (Calbiochem) was stored as 1M in DMSO (Sigma) at −20°C.

### Cell viability assay

HepG2 and Bel7402 were plated into 96-well plates at the density of 1×10^4^ cells/well and cultured for 24h. Celastrol was added to the wells at a final concentration of 0, 0.625, 1.25, 2.5, 5.0 and 10.0μM, while wells with equimolar DMSO were used as controls. After the cells were incubated for 24h, the CCK-8 (Dojindo) solution was added to each well followed by incubation for 40 min to 1 h at 37°C. Absorbance of each well was measured at 450 nm using a spectrophotometer (Tecan). For the time course cell viability assay, HepG2 and Bel7402 cells were treated with DMSO, or 2.5μM celastrol alone, or 2mM TUDCA was added 40 minutes before celastrol. All cell viability assays were performed at least three times.

### Clonogenic survival assay

HepG2 and Bel7402 cells were seeded at 800 cells/well in 6-well plates and dispersed evenly by shaking. Attached cells were treated with celastrol or DMSO control, as indicated. After 1h, the medium was replaced by complete (drug-free) medium and the cultures were grown for 10 days. The colonies were fixed and stained with 0.5% crystal violet in absolute ethanol, then photographed and counted; data are representative of three independent experiments.

### Analysis of cell cycle and apoptosis by flow cytometry

HepG2 and Bel7402 cells were plated at a density of 2×10^5^ cells/well in 6-well plates. The next day, cells were treated with celastrol for 16 h. Cells were harvested and fixed with 70% ethanol at 4°C, overnight. Cells were further incubated with RNase (10μg/μl) at 37°C for 30 min followed by the addition of propidium iodide (5μg/μl) for 3min. The DNA content was evaluated in a flow cytometer (BD FACS ARIAII). The data were analyzed using Modfit software (BD Biosciences). For the Annexing assay, HepG2 and Bel7402 cells were plated at a density of 2×10^5^ cells/well in 6-well plates. Twenty-four hours later cells were treated with different doses of celastrol for 16h. Cells were then harvested and washed twice with pre-chilled PBS and re-suspended in 100μl binding buffer (supplied by the vendor). Cells were stained with Annexin V-FITC and propidium iodide according to the manufacturer's protocol (Thermo Scientific) before analysis by FACScan flow cytometry. All of the experiments reported in the present study were performed in triplicate.

### Transmission electron microscopy observation

Changes in cell ultra-structure caused by celastrol were visualized using transmission electron microscopy (TEM). Autophagy was evaluated by examining autophagosome formation. Briefly, the treated cells were fixed with 4% glutaraldehyde and post-fixed with 1% osmium tetroxide. After being dehydrated in increasing concentrations of alcohol, the cell pellets were embedded in Eponate 12. Representative areas were chosen for ultra-thin sectioning and examined on a TEM at indicated magnification.

### TUNEL assay

For TUNEL assay, after exposure to celastrol for 24h, HepG2 cells were fixed with 4% paraformaldehyde. TUNEL was performed according to the manufacturer's protocol (Roche, 11684817). The cells were then stained with DAPI (Invitrogen) and observed with fluorescence microscopy (Olympus).

### Fluorescence microscopy

Bel7402 cells were seeded on sterilized coverslips, and then were induced with or without celastrol for 24h. Cells were fixed with 4% paraformaldehyde for 30min at RT. Coverslips were blocked and incubated with primary antibodies (anti-LC3B Cell Signaling 1:200 in PBS) overnight at 4°C. Coverslips were incubated with anti-rabbit antibodies (Abcam 150075) conjugated with Alexa Fluor® 647 for 1h at RT. Images were taken using a confocal microscope (Olympus).

### Real-time PCR analysis

1μg of RNA sample was reverse-transcribed to generate cDNA libraries, which were subjected to SYBR green based real-time PCR analysis. Primers used for GAPDH forward: 5`-GGAGCGAGATCCCTCCAAAAT-3` and reverse 5`-GGCTGTTGTCATACTTCTCATGG-3`; for Bip forward 5`-TGACATTGAAGACTTCAAAGCT-3` and reverse 5`-CTGCTGTATCCTCTTCACCAGT-3`; for IRE1 forward 5`-TGCTTAAGGACATGGCTACCATCA-3` and reverse:5`-CTGGAACTGCTGGTGCTGGA-3`; for XBP1 splicing forward 5`-CCTGGTTGCTGAAGAGGAGG-3` and reverse 5`-CCATGGGGAGATGTTCTGGAG-3`; for XBP1 total forward 5`-AGGAGTTAAGACAGCGCTTGGGGATGGAT-3` and reverse 5`-CTGAATCTGAAGAGTCAATACCGCCAGAAT-3`. for ATF4 forward 5`-GCTAAGGCGGGCTCCTCCGA-3` and reverse 5`-ACCCAACAGGGCATCCAAGTCG-3`; for CHOP forward 5`-GGAGCATCAGTCCCCCACTT-3` and reverse 5`-TGTGGGATTGAGGGTCACATC-3`.

### Immunoblot analysis

Total cell extracts or nuclear extracts were separated by 10% or 12% SDS-PAGE and transferred to PVDF membranes. The following primary antibodies were used for immunoblot analysis: anti-GRP78/BiP (Cell Signaling, 3177); anti-IRE1α (Cell Signaling, 3294); anti-p-IRE1 (Abcam, 124945); anti-PERK (ab79483), anti-XBP1s (Cell Signaling, 12782); anti-cleaved PARP (Cell Signaling, 5625); anti-cleaved caspase-3 (Cell Signaling, 9664); anti-LC3B (Cell Signaling, 3868); anti-Beclin1 (Abcam, 207612) and β-actin (Santa Cruz, 70319) at a 1:1000 dilution. Horseradish peroxidase-conjugated secondary anti-mouse or anti-rabbit antibodies (Cell Signaling) were used at a 1:2000 dilution. Detection was performed by using a SignalFireTM ECL Reagent (Cell Signaling, 6883).

### Hepatoma H22-bearing mouse and treatment

4 to 6 weeks old female BALB/c mice (18-20g), were purchased from the Laboratory Animal Research Center of Jilin University (Changchun, China). Animal experiments were conducted according to the Guide for the Care and Use of Laboratory Animals of Jilin University, as approved by the Animal Care committee of Jilin University. BALB/c mouse were inoculated with H22 cells by subcutaneous injection of 2×10^6^ cells in the right flank. One day after implantation of tumor cells, the mice were randomly divided into three groups. One group was administered with corn oil by intraperitoneal injection each day (vehicle control group), and the other groups was treated with celastrol in corn oil (1 or 2 mg/kg/day) by IP injection for 30 days. One day after starting the treatment, the tumors were observed using a small animal *in vivo* optical imaging system (IVIS Spectrum, Caliper Life Sciences, USA) every week after the first treatment, their volume was subsequently calculated. Growth curves were plotted using average relative tumor volume within each experimental group at the set time points. To determine the celastrol effect on the tumor growth, tumor samples were collected at ending of the experiment.

### Statistical analysis

The data are expressed as the mean ± SD. SPSS 21.0 software was used for analysis. All experiments were repeated at least three times. The statistical significance of differences between two groups was assessed using Student's t-test, and P < 0.05 was considered to indicate a statistically significant result. One-way analysis of variance and independent sample t-test were used in the murine syngeneic model to analysis tumor volume and body weight.

## SUPPLEMENTARY MATERIALS FIGURES


