# Supplementary material for: Celastrol induces apoptosis in hepatocellular carcinoma cells via targeting ER-stress/UPR
Source: Oncotarget. 2017 Oct 10;8(54):93039–50. doi: 10.18632/oncotarget.21750 (PMC5696242; doi:10.18632/oncotarget.21750)
Supplement: Supplementary file 1 [file oncotarget-08-93039-s001.pdf]

Celastrol induces apoptosis in hepatocellular carcinoma cells via targeting ER-stress/UPR

SUPPLEMENTARY MATERIALS

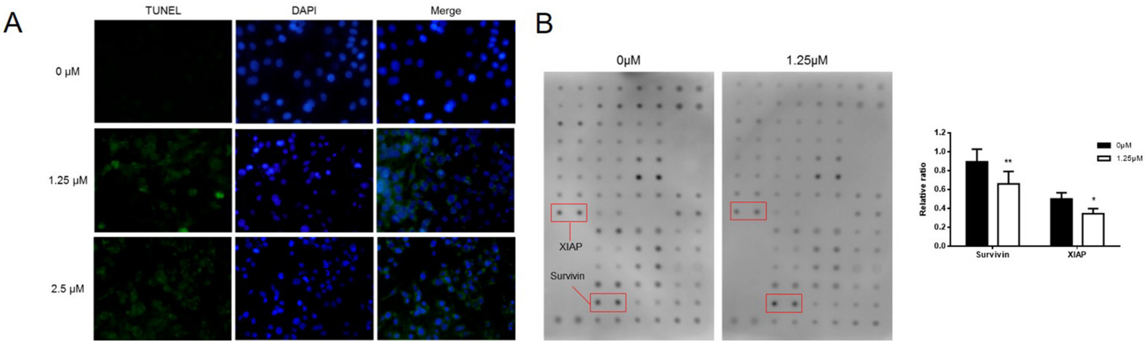

Supplementary Figure 1: Celastrol induced apoptosis in Bel7402. (A) Apoptotic cell death in Bel7402 by celastrol treatment was demonstrated by TUNEL assay, and representative micrographs are shown (magnification, ×200). **(B)** Original figure of Human Apoptosis Antibody array (abcam 134001) and graph of protein expression detected by array. *P<0.05 compared with the 0 µM, student's t-test.

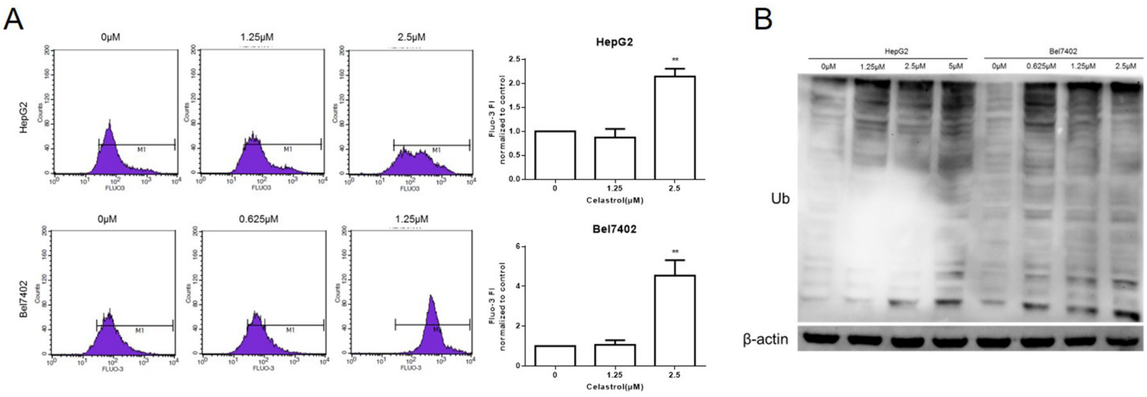

Supplementary Figure 2: Celastrol mediated Ca^{2+} release and increased accumulation of total ubiquitinated proteins in HCC cells. **(A)** HCC cells treated with celastrol for the 12 hours were stained with 2.5 μM Fluo-3 and processed for FACS analysis. Fluo-3 fluorescence intensities (FI) in cells treated with a serial dose celastrol were compared with that of untreated cells and denoted in the graph. **(B)** Immunoblot analysis of total ubiquitinated proteins in whole cell lysates after 16 hours for HCC cells.
